# Application of Teager–Kaiser’s Instantaneous Frequency for Detection of Delamination in FRP Composite Materials

**DOI:** 10.3390/ma14051154

**Published:** 2021-03-01

**Authors:** Adam Gałęzia, Anita Orłowska-Gałęzia

**Affiliations:** 1 The Faculty of Automotive and Construction Machinery Engineering, Warsaw University of Technology, Narbutta 84, 02-524 Warsaw, Poland; 2 Department of Intelligent Technologies, Institute of Fundamental Technological Research, Polish Academy of Sciences, Pawińskiego 5B, 02-106 Warsaw, Poland; aorlow@ippt.pan.pl

**Keywords:** delamination, Teager–Kaiser energy operator, instantaneous frequency, fiber-reinforced composite material

## Abstract

Composite materials are widely used in many engineering applications and fields of technology. One of the main defects, which occur in fiber-reinforced composite materials, is delamination. It manifests itself in the separation of layers of material and the damaged structure once subjected to mechanical loads degrades further. Delamination results in lower stiffness and the decrease of structure’s carry load capability. Its early detection is one of the tasks of non-invasive structural health monitoring of layered composite materials. This publication discusses a new method for delamination detection in fiber-reinforced composite materials. The approach is based on analysis of energy signal, calculated with Teager–Kaiser energy operator, and comparison of change of the weighted instantaneous frequency for measurement points located in- and outside of delamination area. First, applicability of the developed method was tested using simple models of vibration signals, reflecting considered phenomena. Next, the authors’ weighted instantaneous frequency was applied for detection of deamination using signals obtained from FEM simulated response of the cantilever beam. Finally, the methods effectiveness were tested involving real experimental signals collected by the laser Doppler vibrometer (LVD) sensor measuring vibrations of the delaminated glass-epoxy specimens.

## 1. Introduction

Fiber-reinforced polymer (FRP) composite materials have found application in a wide range of engineering structures—from parts of vehicles, through structures like pedestrian bridges to wind turbine blades. The main cause of their popularity is very good strength to mass ratio.

Both, during manufacturing as well as use, FRP structures are subjected to a number of degradation processes, such as cracks or delamination. Degradation can result from loading the structure with force over accepted level, e.g., as a result of impulse, or due to violating construction’s fatigue strength on cyclic load. According to present state of technology, the origination of a defect does not preclude further use of a structure. However, further operation of an object, in accordance with a given level of safety, implies the need for early detection, identification, and localization of the fault as well as controlling fault development. Detection of an early phase of a fault allows to change operation conditions or to plan maintenance and repairs [1,2].

Delamination is the separation of part of a material of FRP which causes local change of its stiffness. Further loading of a structure, will effect, in the growth of delamination area or, in an extreme case, its rapid development causing destruction or irreversible loss of operation ability of a structure, e.g., like in the case of helicopter rotor blade delamination which took place in Israel in 2009 [3]. Due to the importance of this degradation mechanism, as well as the difficulty to detect and localize delamination, a number of diagnostic methods have already been considered by engineers and still new ones are being developed.

Those methods are based on observations of different physical phenomena such as: ultrasonic waves [4,5], acoustic emission [6], thermographic response of the structure excited with the thermal (IR) radiation or external forces [7,8,9], or vibrations [10,11]. Despite there still being some challenges for ultrasonic and thermographic methods [12], these are the leading methods used for inspections of high-risk composite components like, e.g., aerospace structures [6]. They allow to fully identify relatively small delamination, but the qualified operator has to set the test and the inspection process may take many hours to complete [13].

Due to economical and practical reasons, the methods based on observation and analysis of vibrations have gained big popularity, mainly in structural health monitoring applications. It is caused by the fact that the vibration signal can be easily measured by relatively cheap equipment [14]. There is a broad range of measurement technologies, starting from classical transducers like accelerometers [14], through optical fibers [14], piezoelectric strain sensors [15], micro-electro-mechanical systems (MEMS) sensors [16,17], up to touchless measurement methods realized, e.g., by laser Doppler vibrometry [18]. In addition, most frequently, the operating conditions cause the structure to vibrate, opening opportunity to possess diagnostic information without adding additional excitation energy.

Literature presents many techniques, which are dedicated to dealing with vibrational response of a structure and aim for detection and/or identification of the structural damages, such as delamination. A great number of methods are based on inverse problem analysis [19,20]. For example, in [21], the authors presented results of the examination of the three different inverse algorithms for predicting the location and size of delamination: direct solution using a graphical method, artificial neural network (ANN), and surrogate-based optimization. Discussed algorithms have been validated using numerical data generated from the finite element (FE) model of delaminated beams, as well as data from experimental modal analysis conducted on carbon-fiber reinforced polymer beam specimens. It was shown that all the presented algorithms accurately predicted the delamination parameters, when the FE model validation data were used. However, when experimental data were used, the ANN algorithm turned out to be sensitive to the measurements errors.

In [22], the authors showed that delamination can be identified, solving the inverse problem with the use of gradient optimization, when the virtual distortion method is used for modeling of the healthy structure. The delamination identification scheme was formulated by the inverse problem also in [23,24]. Inverse analysis methods are usually very effective in delamination identification. They are capable of finding the exact delamination location, with the use of input data collected by low number of sensors. From the other hand, they are very time consuming, as they need demanding learning process, e.g., in the case of ANN use, or a detailed model of the investigated structure.

Another group of methods identify delamination by the use of so-called damage indicators. The most popular damage indicators are based on time domain signals or modal characteristics extracted from the vibrational signal. The example of such an indicator is the probabilistic delamination indicator (PDI) presented in [25]. PDI is developed on the basis of delamination induced relative frequency change curves and its relationship to mode shapes. It was proved, that the method is capable to detect mid-plane as well as off-mid-plane delamination in laminated composite beams. It was also shown that the proposed method shows two symmetric, potential locations of delamination and because of that, can be used for preliminary tests, preceding the use of, e.g., an ultrasonic apparatus. Additionally, in [26], the method based on the computation of the damage indicators was presented. The authors suggested damage indicators based on the analysis of phase representation of signals. In this approach, the analyzed signal is presented on a phase plane as the Poincare map. The identification and localization of failure in the form of delamination, is performed based on the comparison analysis between representation of signals from structure with and without a failure. Some other damage indicators are depicted in [27].

The methods based on the use of damage indicators are significantly simpler and less time consuming then the inverse analysis methods, but there are still areas that need to be improved. Many indicators exhibit sensitivity problems, need a reference state, and do not provide the possibility of detecting false alarms, reducing their reliability.

Intelligent signal processing-based algorithms can be pointed as the next group of delamination identification methods. An interesting example of signal processing-based identification is presented in [28]. The author proposed a two-step delamination detection and evaluation procedure, which consists of modal shapes extraction and an advanced signal processing algorithm, based on 2D wavelet transform (WT) with B-spline wavelets of fractional order. Another novel testing procedure of that type, based on the feature extraction capability of multi-level wavelet-based processing, is presented in [29]. Intelligent signal processing-based methods are using advanced mathematical tools. They frequently operate as multi step algorithms and require preliminary selection of signal processing parameters.

As the delamination is a very serious damage and can result in catastrophic failure, there is a very high concern in the scientific community in finding the effective methods allowing for its identification. The papers mentioned above represent only a small fraction of the published literature resources in the field, showing the diversity of scientific tools adopted for delamination identification purposes. In the more broad discussion on the vibration-based delamination assessment methods, their potential and classification are a job for a self-contained review article rather than for the introductory part of the research paper; a general conclusion coming from at least few review papers [20,30,31] can be presented as a good state of the art summary. Conclusions can be drawn that all presented techniques have their own advantages and disadvantages and there is no general technique that allows to identify all kinds of delamination in all kinds of structures. In addition, taking into account that most of the methods presented in literature are examined on simple elements like beams or plates, it seems that in the case of more composed objects, the reliable SHM system should use at least a few identification methods. It legitimizes the search for new, complementary methods of delamination identification.

In the context of the above, the proposed method should not be considered as a stand-alone solution, being a perfect remedy, but as one of possible assisting methods in the vibration-based SHM system. Nonlinear dynamics of breathing deformation, observed in the finite element model of the vibrating specimen as well as in experimental tests, points to the need of selecting proper signal processing and analysis methods allowing to detect this phenomena.

The presented approach, is based on the Teager–Kaiser energy operator (TKEO). The analysis of the time domain signal using the Teager–Kaiser energy operator enables observation of transient disturbances of the signal’s instantaneous frequency (IF) [32]. Such disturbances can result from a failure, e.g., crack or delamination of a composite structure. The main assumption underlying the developed method is local, periodic change of stiffness of the tested object due to opening and closing of delamination which will manifest in the change of an instantaneous frequency of vibration. As the change of stiffness in delaminated area occurs within a single period of vibration, the vibration of delaminated beam is characterized by specific half-period fluctuation of instantaneous frequency.

In contrast to many present vibration-based methods, the original TKEO-based delamination detection method does not require knowledge of dynamic behavior of the structure in reference, i.e., healthy state, nor referring to the numerical model. In the considered case of breathing delamination, comparison of instantaneous frequency of vibration signals recorded in measurement points located within and outside delamination allows to create a useful failure indicator.

The paper is organized in the following order: Section 2 discusses disturbance of the vibration signal resulting from opening and closing of delamination. Section 3 presents a simplified model of a vibration signal, in which variation of instantaneous frequency within each half-period of vibration is assumed to result from phenomena of breathing delamination. Section 4 is devoted to discussion on the applicability of selected signal processing methods for detection of considered half-period disturbance of instantaneous frequency. This section also gives some details on the Teager–Kaiser energy operator and introduces the original Teager–Kaiser weighted instantaneous frequency $f_{w}$. The analysis described in this section was performed on the signal obtained using the model described in the previous section. Those works were preliminary activities before the FEM model was built and experimental tests were performed. Section 5 presents results of tests performed with the FE model of fiber-reinforced composite specimens. In contrast to the numerical model from Section 3, the FE model allowed to obtain more realistic behavior regarding amplitude and frequency variations. Laboratory tests of specimen with artificially introduced delamination and their results are presented in Section 6. Section 7 concludes performed research as well as discuss advantages and drawbacks of the Teager–Kaiser weighted instantaneous frequency $f_{w}$.

## 2. Breathing Delamination Phenomena

**Figure 1:**
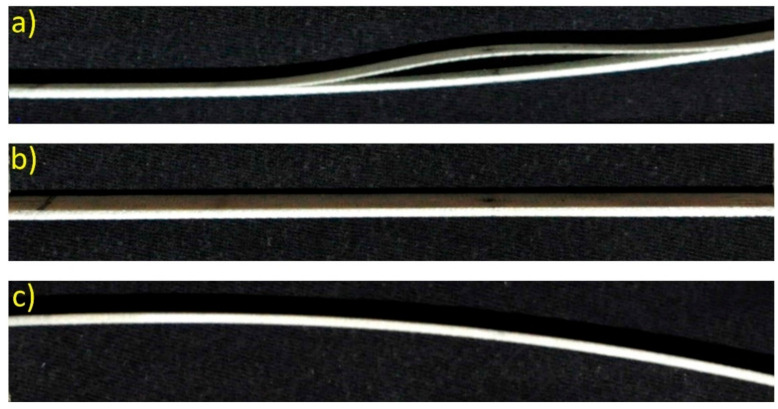
Breathing delamination of the beam structure during vibration: (**a**) opening of delamination when the beam tip goes up; (**b**), beam in equilibrium; (**c**) closing of delamination when the beam tip goes down.

Present-day publications [33,34] as well as numerical models discussed in Section 4 and results of experimental tests presented in Section 5, indicate that in specific mode shapes of delaminated structure, relative motion of the delaminated layer can be observed. This phenomenon is known as opening and closing of delamination, or breathing delamination (Figure 1) and it causes fluctuations of stiffness [35] in the delaminated structure. As a result of the variation of stiffness, the frequency of vibrations undergoes instantaneous changes. It can be assumed that delamination reveals itself in a specific signal pattern with uneven half-periods. During the opening of delamination (Figure 1a), the structure has lower stiffness, which is reflected in lower eigenfrequency. When delamination is closed (Figure 1c), the stiffness of a specimen is close to the stiffness of an undamaged structure. As a result, eigenfrequency is higher compared to the situation when delamination is opened. During excited vibrations, e.g., in first mode, for a single period of excitation, part of the structure’s motion takes place for opened delamination, while the other part takes place for closed delamination. However, the complete period of the structure’s motion is consistent with excitation frequency.

## 3. Simplified Model of Vibration Signal

To reflect nonlinear dynamic behavior of the structure with breathing delamination and to perform preliminary analysis, the dedicated numerical model of a vibration signal was created. The model must be considered simplified because it is not reflecting any particular breathing delamination of any specific structure. Additionally, it assumes that change of instantaneous frequency takes place in a contiguous way without rapid transitions.

For the preliminary tests, it was assumed that half-periods of the created signal will differ by 2 Hz and instantaneous frequency will continuously vary from 9 to 11 Hz and back. In the first approach, indicated as Model 1, the numerical model was created by combining half-periods of two single harmonic signals with different frequencies using the cubic spline interpolation function $S\left(t\right)$ [36], smoothly connecting sine half-periods. Equation (1) describes single period T of the modeled signal:

(1) $$x{\left(t\right)}_{Model1}=\left\{\begin{array}{c} A\cdot sin\left(2\pi \cdot f_{1}\cdot t\right)\;\;\;\;\;\;\;\;\;\;\;\;\;\;\;\;\;\;\;\;\;\;for\;t\;\epsilon \;\left(0.1T,0.45T\;\right)\\ A\cdot sin\left(2\pi \cdot f_{2}\cdot t\right)\;\;\;\;\;\;\;\;\;\;\;\;\;\;\;for\;t\;\epsilon \;\left(0.625T,0.925T\right)\\ S\left(t\right)\;for\;t\;\epsilon \;\langle 0.925T,0.1T\rangle \;and\;t\;\epsilon \;\langle 0.45T,0.625T\rangle \end{array}\right..$$

To build the numerical signal, the upper half-period of 9 Hz waveform and the bottom half-period of 11 Hz waveform were joined without interval. Due to different curvature of half-periods, the connection point was smoothed with spline interpolation. Due to spline smoothing, the zero-crossing point is shifted from the original zero-crossing of 9 and 11 Hz waveforms. In the $x{\left(t\right)}_{Model1}$ signal, all connection points of half-periods were smoothed, while the beginning and the end of the signal were modified to keep the same curvature of half-periods.

The Model 2 ($x{\left(t\right)}_{Model2}$) is created by combining two harmonic signals with appropriately defined amplitudes $A_{1}$ and $A_{2}$, according to the following analytical Equation (2):

(2) $$x{\left(t\right)}_{Model2}=A_{1}\sin\left(2\pi \cdot f\cdot t\right)+A_{2}\sin\left(2\pi \cdot 2\cdot f\cdot t\right).$$

**Figure 2:**
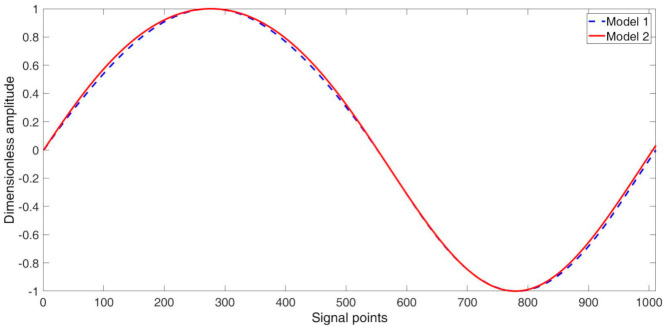
Comparison of waveforms from models.

The selection of amplitude ratio $A_{1}/A_{2}$ as well as value of frequency f allowed to correctly model the non-symmetry of the signal. Figure 2 presents the comparison of the single period for both models with the assumption of representation of the signal with 9 and 11 Hz half-periods.

Main differences between the models are related to spline interpolation smoothing used in Model 1. The smoothing causes modification of part of the curvature of the waveform as well as the shifting of the beginning of the consecutive full period of the signal. Because the signal created by the use of the Model 2 does not suffer from those drawbacks, it was used in further analysis. Next, the section refers only to use of the $x{\left(t\right)}_{Model2}$ signal and it will be denoted as $x\left(t\right)$.

## 4. Detection of Frequency Fluctuation

The section discusses application of three different tools for detection of instantaneous change of the signal frequency. The signal $x\left(t\right)$ (Equation (2)) was analyzed using: spectral analysis, Hilbert transform-based demodulation and the Teager–Kaiser energy operator-based demodulation. The mathematical principia of the presented methods as well as results of the conducted analysis were presented in the next subsections.

### 4.1. Spectral Analysis

**Figure 3:**
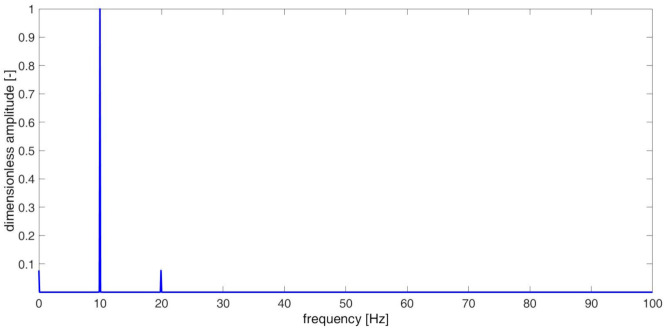
Spectrum of signal $x\left(t\right)$.

Spectral analysis, which is based on the Fourier transform mathematical theorem, is one of most popular tools used in signal analysis [37], e.g., in machines’ condition monitoring. The method allows for decomposition of a time series into harmonic oscillations and represents its amplitude and phase spectrum. The results of the numerical spectral analysis are strongly dependent on the duration of the investigated signal. It is commonly used in measurement practice, which in case of multi-harmonic signals, at least few fundamental periods are analyzed, in order to correctly grasp all components of interest. The result of spectral analysis, conducted on the signal $x\left(t\right)$, is presented in Figure 3. The analyzed signal consisted of 30 complete periods.

The spectrum has 3 harmonic components: DC component, main component with frequency 9.95 Hz, and its second harmonic—19.9 Hz. The frequency of the main component results from the period’s duration of the analyzed signal, which is composed of two different half-periods. Investigating structure, for which it is not possible to refer to the healthy state, the frequency observed in spectrum can be interpreted as modal frequency of the undamaged structure. The spectrum reveals no information regarding deviation of frequency.

### 4.2. Hilbert Transform Demodulation Analysis

The demodulation based on Hilbert transform is well established in the engineering community and found application in many tasks such as, for example, bearing diagnostics [2]. It allows for identification of modulation phenomena and, particularly, for estimation of instantaneous frequency [38].

The complex sum of the analyzed signal $s\left(t\right)$ and its Hilbert transform $\hat{s}\left(t\right)$ creates the analytical signal $\tilde{s}\left(t\right)$ defined as Equation (3):

(3) $$\tilde{s}\left(t\right)=s\left(t\right)+j\hat{s}\left(t\right).$$

The analytical signal allows to estimate envelope (Equation (4)) and instantaneous frequency (Equation (5)) of the analyzed signal:

(4) $$\left|\tilde{s}\left(t\right)\right|=\sqrt{s^{2}\left(t\right)+{\hat{s}}^{2}\left(t\right)},$$

(5) $$\phi \left(t\right)=\arg\{\tilde{s}\left(t\right)\}=arctg\frac{\hat{s}\left(t\right)}{s\left(t\right)}.$$

In the considered case of the nonlinear signal with half-period frequency variation, the Hilbert transform demodulation does not allow to properly detect changes occurring in the signal. According to this approach, the investigated signal has weak amplitude and frequency modulations. The spectrum of the obtained instantaneous frequency signal reveals existence of the 9.95 Hz component as well as the contribution of its higher harmonics.

**Figure 4:**
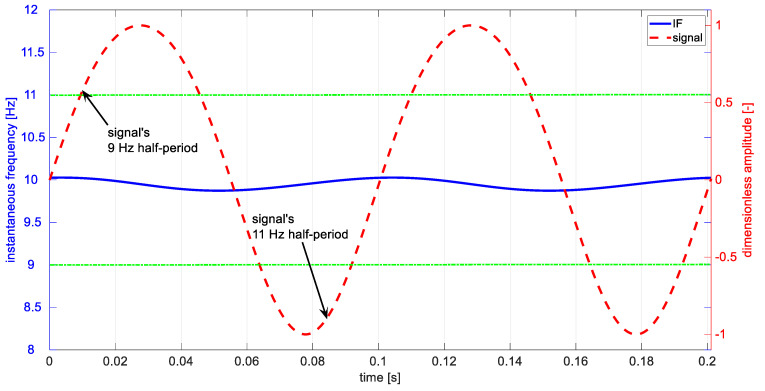
Analyzed signal (red), and time-domain fluctuation of Hilbert demodulation instantaneous frequency (IF) (blue). Green horizontal lines indicate values of the IF expected for the considered case.

The modeled discrepancy of frequency is not correctly represented in the instantaneous frequency (Figure 4). The Hilbert demodulation indicates that instantaneous frequency has small variations around mean value equal to 9.95 Hz. The extreme values of the obtained IF occur for instants of transition of half-periods. Hilbert transform demodulation results do not point to the frequencies related to half-periods and the same does not identify well enough the breathing delamination symptoms.

### 4.3. Signal Processing Using the Teager–Kaiser Energy Operator

The Teager–Kaiser energy operator is a differential operator and was presented in [39] while its properties were discussed in [40,41,42,43]. Many successful applications of TKEO and related measures in condition monitoring of gears and bearings can be found in publications [44,45,46]. One of the features of TKEO is that it calculates the signal’s energy point-by-point. As a result, TKEO is more sensitive to transient changes occurring in signal. On one hand, it is sensitive to noise, but on other hand, it allows for easier, comparing to solutions based on, e.g., Hilbert transform, detection of instantaneous changes in the signal’s amplitude or frequency, e.g., such as transient changes of IF caused by breathing delamination.

Teager–Kaiser energy $E_{TK}\left(t\right)$ is the time-domain signal obtained by the operator $\Psi \left(s\left(t\right)\right)$ (Equation (6)) acting on the analyzed signal $s\left(t\right)$:

(6) $$\Psi \left(s\left(t\right)\right)={\dot{s}}^{2}\left(t\right)-s\left(t\right)\ddot{s}\left(t\right).$$

In [41], the authors presented a compact and easy to implement form of the TKEO for discrete signals (Equation (7)):

(7) $$\Psi_{d}\left(s_{n}\right)=s_{n}{}^{2}-s_{n-1}s_{n+1}.$$

For a certain class of AM-FM signals, described as (Equation (8)):

(8) $$s\left(t\right)=A\left(t\right)\cos\left(\omega \left(t\right)t\right),$$

it can be shown that (Equation (9)) [47]:

(9) $$E_{TK}\left(t\right)=\Psi \left(s\left(t\right)\right)\approx A{\left(t\right)}^{2}\omega {\left(t\right)}^{2}.$$

Extending this concept, the TKEO (Equation (6)) can be applied for determination of the envelope (Equation (10)) and instantaneous frequency (Equation (11)):

(10) $$A^{2}\left(t\right)=\frac{\Psi {\left(s\left(t\right)\right)}^{2}}{\Psi \left(\dot{s}\left(t\right)\right)},$$

(11) $$\omega^{2}\left(t\right)=\frac{\Psi \left(\dot{s}\left(t\right)\right)}{\Psi \left(s\left(t\right)\right)}.$$

The demodulation procedure based on Equations (10) and (11) is known as the energy separation algorithm (ESA) and was presented in [43,48]. Discussion on properties of the algorithms, both for continuous (CESA) and discrete (DESA) signals is presented in [47].

It is important to emphasize here, that because in the general case, the base formula (9) allows for determination of approximate value of Teager–Kaiser energy, Equations (10) and (11) are accurate for harmonic signals only. In the case of signals with AM and FM modulations, both for CESA and DESA, the additional high frequency components arise in related Teager–Kaiser energies. In consequence, some additional conditions have to be imposed on the signal to minimize discrepancy, such as [49]: the analyzed signal is changing slowly, in relation to sampling frequency and the modulation bandwidth and value are significantly smaller from the carrier frequency.

Several DESA algorithms exist [48] and although all known TKEO-based demodulation algorithms were tested for the analyzed signal (Equation (2)), the DESA-2 algorithm was chosen as giving the best results with lowest errors. According to DESA-2 [48], instantaneous frequency is defined by Equation (12) and envelope by Equation (13):

(12) $$\omega_{n}\approx \frac{1}{2}arccos\left[1-\frac{\Psi_{d}\left(x_{n+1}-x_{n-1}\right)}{2\Psi_{d}\left(x_{n}\right)}\right],$$

(13) $$A_{n}\approx \frac{2\Psi_{d}\left(x_{n}\right)}{\sqrt{\Psi_{d}\left(x_{n+1}-x_{n-1}\right)}}.$$

One must be aware of the limitations of this approach. The vibration signal coming from breathing delamination is not an AM-FM class signal and does not fulfill the conditions allowing for proper demodulation. As a result, it is not possible to fully correctly estimate changes occurring in the signal. The value of the instantaneous frequency is overestimated. Additionally, as [50] discusses, mutual influence of modulation phenomena can arise. In case of the considered signals, in order to improve obtained estimation results, the Teager–Kaiser weighted instantaneous frequency $f_{w}$ is analyzed (Equation (14)):

(14) $$f_{w}=\frac{\omega_{n}\cdot A_{n}}{2\pi }.$$

**Figure 5:**
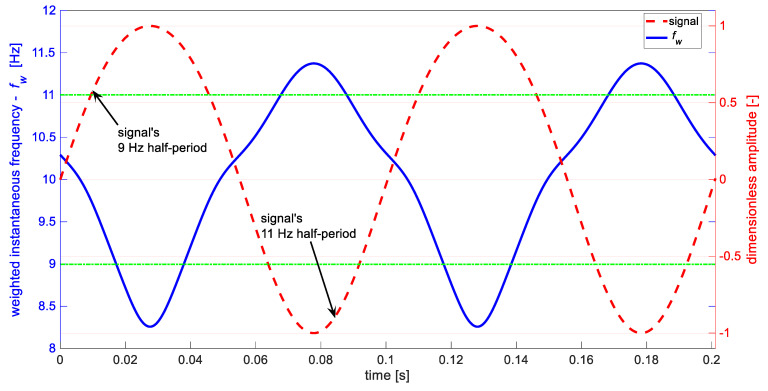
Analyzed signal (red) and calculated weighted instantaneous frequency (blue). Green horizontal lines indicate values of the IF expected for the considered case.

Figure 5 presents part of the modeled signal x(t) and its Teager–Kaiser weighted instantaneous frequency $f_{w}$. The periodic changes of weighted instantaneous frequency $f_{w}$ (blue) are correlated with signals half-periods (red). However, due to imperfection of the used signal’s model $x\left(t\right)$ (Equation (2)), resulting from superposition of harmonic components, the transition between half-periods is not smooth, causing inflections seen in $f_{w}$. The mentioned above limitations of the DESA-2 algorithm cause the method to give acceptable quantitative estimates of weighted instantaneous frequency—the lower (8.26 Hz) as well as higher frequency (11.37 Hz) differ from the modeled parameters (9 and 11 Hz) with an acceptable margin. The presented IF estimation method gives good qualitative results, indicating the existence of breathing delamination symptoms. In contrast to the Hilbert transform approach, the extreme values of Teager–Kaiser weighted instantaneous frequency are concurrent in time with half-periods of the signal. Despite the divergence of the values, the method can find application in practice because it reveals existence of phenomena of interest.

Comparison of analyzed methods clearly show, that only the one based on TKEO demodulation allowed for estimation of modeled changes of IF and revealed phenomena on interest. The spectrum analysis as well as Hilbert demodulation did not make it possible to identify variation of instantaneous frequency.

### 4.4. Estimation of Instantaneous Frequency of Decaying Signal

To define applicability of the described Teager–Kaiser weighted instantaneous frequency (Equation (14)), a number of test cases were investigated. Both steady-state and decaying signals (Equation (15)) were analyzed:

(15) $$x_{decaying}\left(t\right)=A_{d}{}^{-t}\cdot x\left(t\right),$$

where: $x\left(t\right)$—the signal $x{\left(t\right)}_{Model2}$, $A_{d}$—amplitude decaying factor, t—time.

The reason for performing such tests was the higher ease of FE modeling of the structure with breathing delamination producing decaying signals, which were later used as input data for analysis of the method effectiveness in the case of the cantilever beam with delamination (Section 5.4). However, during laboratory measurements of structure, described in Section 6, vibrations with steady amplitude were excited due to technical ease of the experiments. The presented research task ensued to validate the TKEO method for different modeled signals.

Signals with amplitude decaying factor from 1 (no decaying) to 0.1 (fastest decaying) were analyzed to identify whether decaying increases estimation error. For each considered case, the investigated signal was limited to the time range from the beginning till the moment when the signal’s absolute envelope reached the value of 0.2. Statistical parameters for each case, including the signal’s duration, are presented in Table 1. The obtained results differ from the assumed values—11 and 9 Hz, but it is possible to observe the deviation of instantaneous frequency related to the half-periods duration.

The quality of the instantaneous frequency estimation was strongly related to the time length of the signal taken to the analysis. For the signals with low values of $A_{d}$, the calculated weighted instantaneous frequencies $f_{w}$, had higher estimation errors. From performed tests, a conclusion can be made that although the estimation of instantaneous values of frequency becomes less correct with the increase of the decaying factor, still the phenomenon of frequency variation can be observed. Additionally, it is worth to recall that even for signals with short duration, the DESA-2 based approach allowed to perform useful analysis.

**Figure 6:**
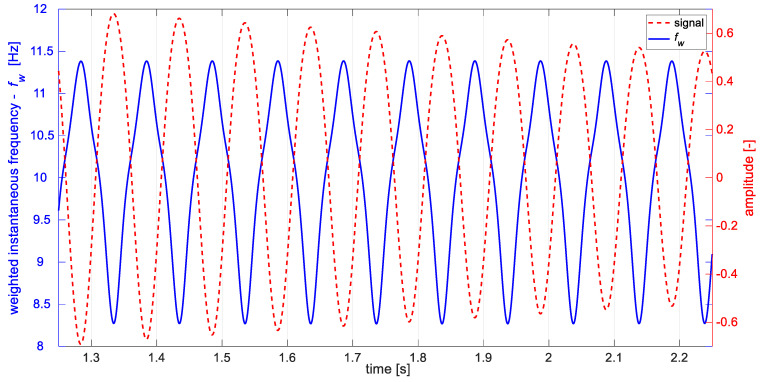
Part of the decaying signal (red) $A_{d}=0.8$ and estimated weighted instantaneous frequency (blue).

Figure 6 presents part of the decaying signal (Equation (15)) and change of its weighted instantaneous frequency $f_{w}$. Similar to the case of signal with steady amplitude, one can observe periodic changes of estimated instantaneous frequency corresponding to the signal’s half-periods.

## 5. Estimation of Instantaneous Frequency of Signal from FEM Simulation

### 5.1. General Description of Modeling of the Delaminated Beam

**Figure 7:**
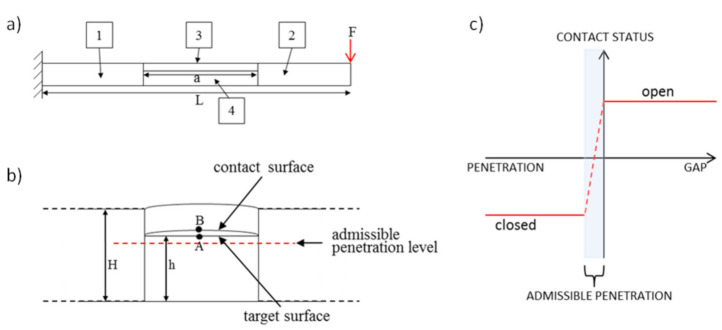
Scheme of the beam parts connections (**a**), zoom of part of the beam with delamination (**b**), contact status definition with penalty-based method (**c**).

The finite element modeling for simulation of a delaminated beam behavior was performed with the use of the commercial finite element software ANSYS. The delaminated beam was composed of four blocks, as it is presented in Figure 7a. The longer inner edge of the block number 3 was modeled by the sinusoidal function. That allowed to reflect realistic crack conditions [51]. The adjoining edges of the blocks, numbered consecutively as 1, 3, 4 and 2, 3, 4, were connected by the constraints imposed on nodal displacements *ux* (horizontal axis) and *uy* (vertical axis). Besides, the CF boundary conditions scheme was applied to the whole delaminated beam, meaning that the beam was fixed in one end. The standard surface-to-surface contact interaction introduced between two faces of the delamination, allowed neither penetration nor separation among the sublaminate structures.

Four-nodal plane elements with a plane strain condition defined in the width direction were used. This is a rather rarely used practice in analysis of beams upon out of plane loading, but this approach was verified by the comparison of the eigenvalues obtained from the discussed model with the eigenvalues of full 3D model. There were no meaningful frequency differences for the first three modes for considered geometry. The use of four-nodal plane elements allows to reduce time cost of computation in comparison to use of 3D brick elements. The additional asset is, that those elements allow to create more realistic conditions at the delamination tips than standard beam elements.

### 5.2. General Description of the Contact Modeling

As delamination splits part of the beam into two distinct segments, which are connected in the delamination tips and can come into interaction during the vibrations, the additional boundary conditions, called contact conditions [52,53,54], have to be fulfilled for each time step of numeric simulation. In ANSYS software, those conditions are defined via the special type of contact elements, appropriate for specific types of contact problems. The type used in the presented simulation, was 2D surface-to-surface contact element. The pair-based contact definition was chosen for this purpose, which means that one of the delamination boundaries was established as the so-called target surface, while the other one as contact surface. Both surfaces are specified in Figure 7. Since the part situated above the delamination (for the purpose of publication called upper sublaminate) is thinner and more flexible than the opposite part, the elements which represent contact surface (dedicated rather for deformable body) were overlaid on the exterior of upper surface. Similarly, the elements which represent target surface and are applicable for rigid as well as flexible bodies, were overlaid on the exterior of lower surface (called lower sublaminate), which is thicker and less flexible.

To enforce maintaining the constraints in contact modeling, the penalty method was used with contact status defined as it is shown in Figure 7c. In this method, the contact force is the function of the penetration distance and some degree of penetration is involved. The computation algorithm used in simulation identified separation and penetration of layers based on the distance between contact surfaces of delamination. For separation, the distance between the layers is positive and as a result, the contact force, used in equations of motion, equals zero. For penetration, the distance has negative value and as a result, the contact force (Equation (16)) is added to equations of motion. The normal contact force $F_{n}$ is defined in the penalty method by the use of so-called normal contact stiffness $k_{n}$ and user-defined admissible penetration $x_{p}$:

(16) $$F_{n}=k_{n}\cdot x_{p}.$$

The value of contact stiffness $k_{n}$ was as small as possible, but also high enough to prevent penetration. Friction was neglected.

The number of equilibrium equations in this method, for most of the cases, is much smaller compared to other methods and the time of the computation can be shortened.

### 5.3. Case Study for 40% Delamintion

For the purpose of demonstrating the breathing delamination effect, transient analysis were performed with the initial condition imposed as the displacement field. Those conditions recreated deformation of the beam, caused due to application of static, perpendicular force in the beam tip. Delamination of the length a equal to 40% of the total beam length L, with the thickness-wise location equal 75% of the beam’s height H, localized in the middle of the beam, was analyzed. The beam material was assumed as anisotropic and its properties are presented in Table 2.

**Figure 8:**
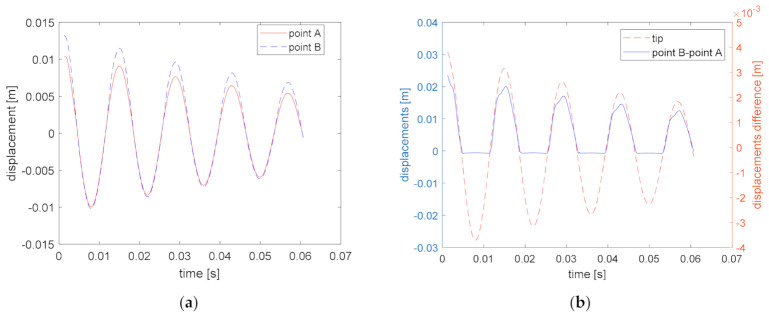
Displacements evolution in points A and B for the case of free vibrations (**a**), the difference of displacement of point A and B and normalized tip displacements (**b**).

Figure 8a shows time evolution of the vertical displacements in points A and B, for the discussed case. As it is shown in Figure 7b, points A and B are localized in the middle of the delamination area, but point A belongs to the lower sublaminate, while point B belongs to the upper sublaminate. When the beam goes downwards, delamination is closed and displacements of the points A and B are practically the same, while when the beam goes upwards, delamination surfaces remain detached from each other and point B reaches a higher amplitude than point A. Figure 8b allows to compare the difference of the vertical displacements in points A and B with the tip displacements.

### 5.4. Analysis of FEM Signals

The numerical FE model described in Section 5.2, was used to simulate the vibrations of the beam with different delamination lengths. For each case, delamination was located in the middle of the beam’s length and at 75% of the beam’s height. The ratios of delamination size with regard to specimen length $x_{a}/L$, ranging from 0.2 to 0.6, were examined. For each case, the displacements time signal from two points: the one located in the middle of the beam and the second located in its tip, were collected and analyzed.

**Figure 9:**
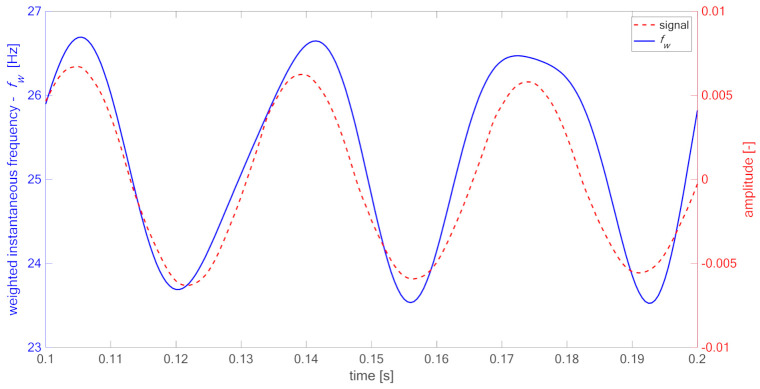
Part of the decaying signal (**red**) from the FEM simulation and its Teager–Kaiser weighted instantaneous frequency (**blue**), case $x_{a}/L=0.4$.

To obtain the band-limited signal, low-pass filtration was applied in order to remove influence of higher frequencies. As it can be seen in Figure 9 for the case of $x_{a}/L=0.4$, periodic variations of Teager–Kaiser weighted instantaneous frequency occur due to the opening and closing of delamination. The same phenomena are observed for all investigated cases. Table 3 presents calculated statistical parameters for estimated $f_{w}$ in relation to $x_{a}/L$ ratio for the node located in the delamination area (point “delam”) and the node located at the free end of the specimen (point “tip”). For big delamination, i.e., $x_{a}/L=0.5$ and $x_{a}/L=0.6$, the difference between lowest and highest $f_{w}$ identified in the signal exceeds 5 Hz.

**Figure 10:**
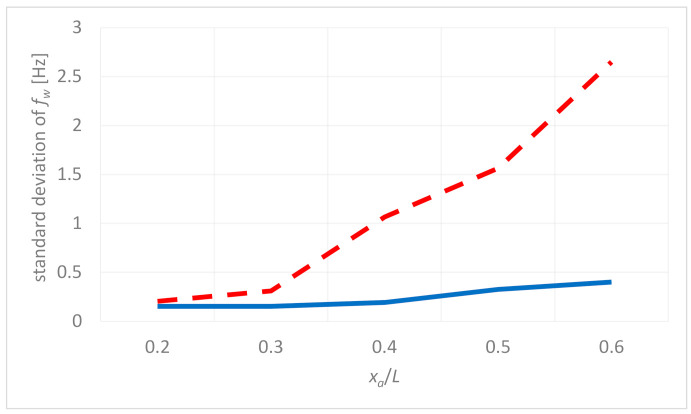
Standard deviation of $f_{w}$ for the delamination center (**red**, **dashed**) and free-end (**blue**).

It can be observed that the deviation of $f_{w}$ for given $x_{a}/L$ ratio is significantly smaller in the case of “tip” node comparing to the “delam” node located over the delamination. For big delamination, the frequency deviation can also be observed in the point located at the free end. This is related to the influence of large delamination on dynamical behavior of the whole structure. Figure 10 shows comparison of standard deviation of weighted instantaneous frequency $f_{w}$ for the point located in the delamination area (red, dashed) and the free end of the FE model (blue), revealing significant difference between points located on the same structure. As shown, the point in the delamination area demonstrates higher difference of half-period frequencies then the point located outside this area, which gives the prospect not only for the detection of delamination existence but also identification of its size.

For validation purposes, the signals were also analyzed with the Hilbert transform demodulation approach. In contrast to the TKEO-based method, which allowed to identify variation of $f_{w}$ between successive half-periods for all tested signals, the Hilbert transform demodulation approach revealed 2.5 Hz variation of the IF only for the biggest delamination ($x_{a}/L=0.6$). For other tested cases, the IF was not diagnostically informative. For this reason, results presented in the consecutive part of the publication are limited to the ones obtained with the estimated Teager–Kaiser weighted instantaneous frequency approach.

## 6. Experimental Validation of Teager–Kaiser Weighted Instantaneous Frequency

### 6.1. The Specimens

**Figure 11:**
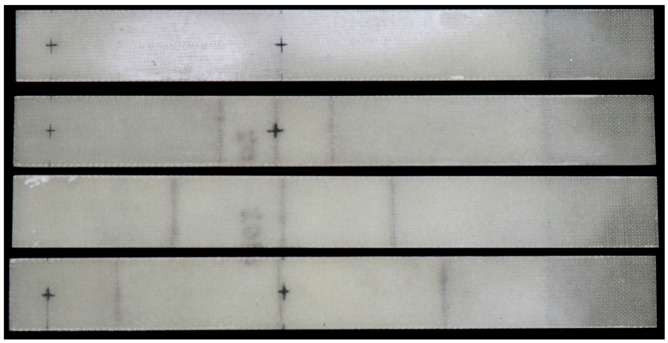
The specimens, from top to bottom respectively: 00, 20, 40, 60.

**Figure 12:**
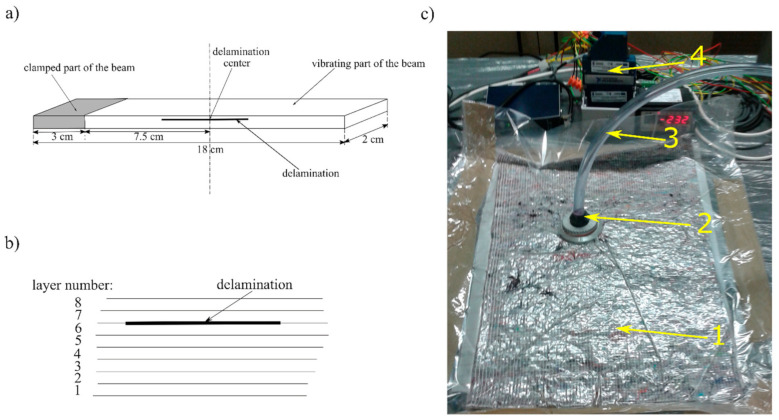
Specimens’ geometry and delamination placement (**a**), general scheme of layer arrangement for test sample (**b**), experimental stand during samples preparation (**c**), where: 1—form with constituent materials in vacuum-bag; 2—vacuum connector; 3—vacuum hose; 4—data acquisition system controlling process parameters.

For preparation of the tests specimens, an 8th layered composite plate was manufactured from bidirectional E-glass fabric (Saerbeck, Germany) of basis weight 163 g/m^2^ and epoxy resin ARALDITE LY3505 (Woodlands, TX, USA) with hardener XB 3403 (Woodlands, TX, USA), by the use of vacuum-bag method. During the hand lay-up of the fabric, three 0.0001 m thick Teflon inserts of different dimensions were placed between the 6th and 7th layer of the composite. Four individual test specimens of nominal dimension 0.18 m × 0.02 m × 0.0014 m were cut from the laminate after resin solidification and the inserts were removed after cutting out specimens. The inserts allowed to create a partially separated layer with controlled length and edges. The created delamination span through the whole width of the beams. Throughout vibration tests, specimens were clamped along one of the tips with 0.03 m distance and active length was equal to 0.15 m. The center of delamination was located at 0.075 m from the free end, i.e., in the center of the active part of the specimen. During the experiment, four specimens were tested: one without delamination ($x_{a}/L=0$, further indicated as 00) and three with different ratios of delamination length $x_{a}$ to active length L, respectively: $x_{a}/L=0.2$ (indicated as 20), $x_{a}/L=0.4$ (indicated as 40), and $x_{a}/L=0.6$ (further indicated as 60). The photo of specimens is presented in Figure 11, while the specimens’ geometry, layer arrangement and the photo of the experimental stand during the samples preparation are shown in Figure 12.

### 6.2. The Experimental Setup

**Figure 13:**
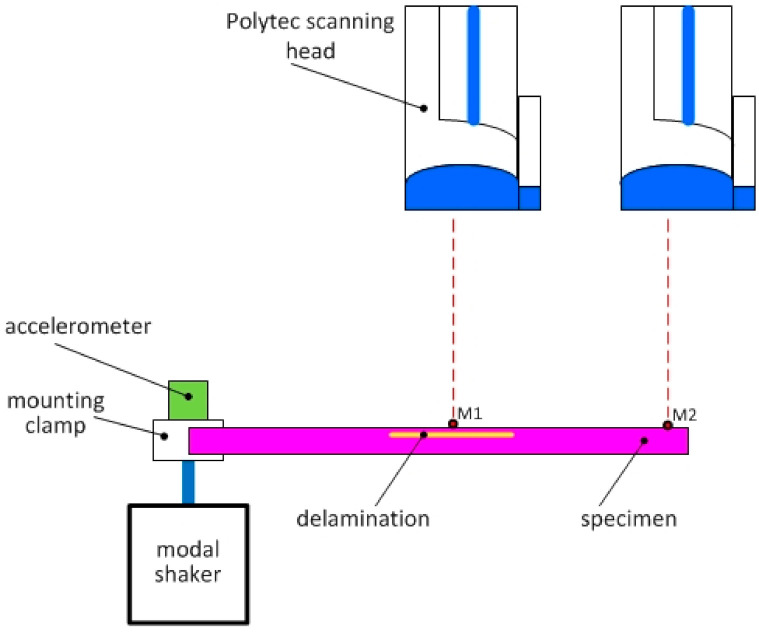
Experimental setup—scheme with measurement points M1 and M2.

The schematic diagram is presented in Figure 13. The tested specimen was placed in a mounting clamp attached to the armature of the modal shaker ModalShop 2100E11. The modal shaker and the mounting clamp excited vertical vibrations which caused bending modes of specimens. The specimens had the CF boundary conditions—one end of the tested beam was fixed and the other was vibrating freely. During all experiments, bolts of the mounting clamp were screwed with force of 7 Nm using torque wrench. The Bruel&Kjaer 4507-B-004 accelerometer sensor was mounted on top of the mounting clamp for reference measurement of excitation signal. Vibrations of the specimen were recorded using Polytec PSV-400-3D laser Doppler vibrometer, set in “single measurement head” configuration. The LDV sensor measured bending vibrations in direction perpendicular to beams surface, in two measurement points: one located in the center of delamination area (indicated M1) and second located at the free end of the specimen (indicated M2). To record signals from both measurement points, the scanning head was shifted between measurement locations using special frame, allowing for repetitive setting of the head. The shaker excited the specimen to vibrate according to control signal generated by the LDV’s inner generator.

### 6.3. The Measurements

The measurements were conducted in two steps. In the first one, all specimens were excited with pseudo-random noise signal and modal analysis was performed in the range (0, 2000) Hz. This band covered the first five bending modes of the specimens. The results are presented in Table 4. The modal analysis reveals differences in modal frequencies between specimen 00 and specimens with delamination. However, the monotonic trend is observed only for third and fifth modal frequency. Direct application of the modal frequencies for structural health monitoring requires that modal frequencies of an undamaged structure are known in advance, which allows to identify the difference resulting from failure [55]. This method does not allow for the localization of a failure. To identify the localization of delamination, methods such as mode shape curvature could be used [56].

It must be pointed out, that in the case of the first two modes, the differences between modal frequencies of the individual specimens can be related to its weight and width variation, as well as influence of mounting errors. First modal frequencies might not be an objective criterion for technical state assessment.

In the second step, the specimens were excited with the harmonic signal with the frequency equal to the identified first modal frequency. During the test of a single specimen, the Polytec vibrometer recorded signals from measurement points: M1 (center of specimen) and M2 (free end of specimen—out of delaminated area). For each measurement point, the vibrometer recorded the simultaneously time domain vibration signal of specimen and reference vibration of mounting clamp. The sampling frequency was equal to 12,800 Hz. Duration of the recorded waveforms was 5.12 s.

Because fluctuation of instantaneous frequency, discussed in the Section 3 and Section 5, manifests strongly in displacement signal, therefore, velocity signals, recorded by the laser vibrometer, were integrated using numerical procedure from the Matlab environment [36]. Although the excitation signal was harmonic, the mechanical system of the shaker, as well as the environment, produced a measurement noise. The presence of noise in analyzed signals decreases quality of results obtained using the Teager–Kaiser energy operator. To reduce the influence of the noise and increase the signal-to-noise ratio, each measurement signal was time synchronously averaged (TSA). This procedure used zero-crossing of rising edge of the reference signal. Averaging caused reduction of measurement signals to realizations with duration of 10 full periods of the reference signal. For such averaged signals, $f_{w}$ was calculated and further analyzed.

### 6.4. Experimental Results

TSA displacement signals from measurement points M1 and M2 of the successive specimens as well as reference signal, measured by the sensor located on mounting clamp, were processed with the use of the DESA-2 algorithm to obtain $f_{w}$.

**Figure 14:**
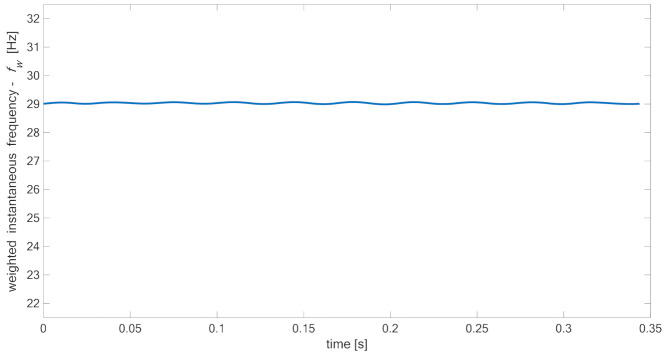
Weighted instantaneous frequency $f_{w}$ of reference signal (specimen 00).

The weighted instantaneous frequency of reference signal was oscillating close to the assumed frequency of the excitation signal as it is presented in Figure 14. The small fluctuations might result from work conditions of the used modal shaker, which is rather dedicated to heavy structures.

**Figure 15:**
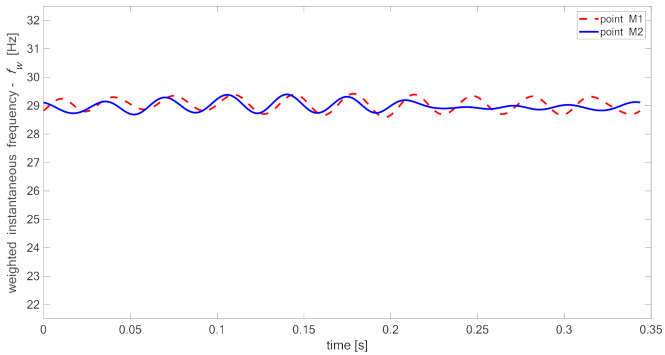
Teager–Kaiser weighted instantaneous frequency for sample 00.

**Figure 16:**
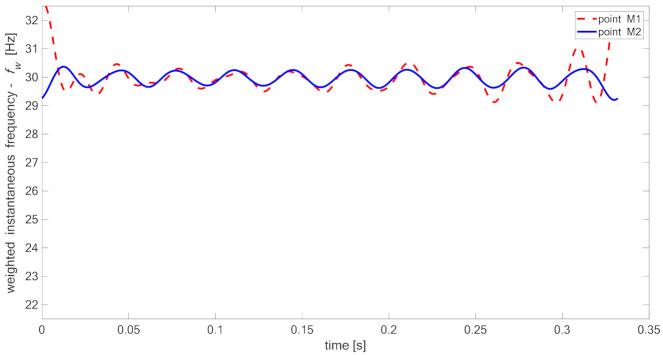
Teager–Kaiser weighted instantaneous frequency for sample 20.

**Figure 17:**
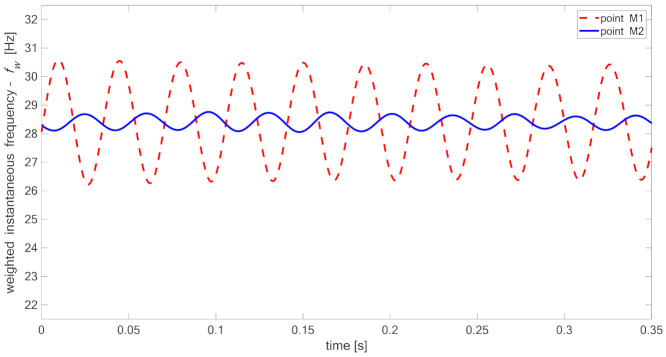
Teager–Kaiser weighted instantaneous frequency for sample 40.

**Figure 18:**
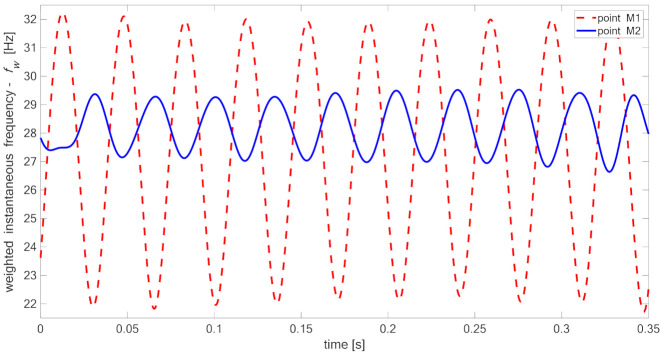
Teager–Kaiser weighted instantaneous frequency for sample 60.

Figure 15, Figure 16, Figure 17 and Figure 18 present time fluctuations of Teager–Kaiser weighted instantaneous frequency $f_{w}$ related to M1 and M2 measurement points for samples 00, 20, 40, and 60 respectively. The consistent scale was used for the sake of comparison. Each figure presents deviation of $f_{w}$ for both measured signals. For the sample without delamination, the deviation of the weighted instantaneous frequency for both points is similar and is close to the deviation of $f_{w}$ of the reference signal. It was observed that with the increase of delamination size, the fluctuations of the weighted instantaneous frequency from point M2 increase, but still remain smaller than the fluctuations measured in the point M1, which is located directly above the delamination.

For comparison of the presented signals and evaluation of delamination, statistical parameters can be used such as: standard deviation (Equation (17)), coefficient of variation (Equation (18)), or values span (Equation (19)) of the weighted instantaneous frequency $f_{w}$:

(17) $$S=\;\sqrt{\frac{1}{N-1}\sum_{i=1}^{N}{\left|x_{i}-\bar{x}\right|}^{2}},$$

where $x_{i}$—successive samples of signal, N—number of samples of signal, $\bar{x}$—mean value of signal $\bar{x}=\frac{1}{N}\cdot \sum_{i=1}^{N}x_{i}$.

(18) $$V=\frac{S}{\bar{x}},$$

where $\;\bar{x}\;$—mean value of signal, S—standard deviation (17).

(19) $$D=\max\left(x\right)-\min\left(x\right).$$

Values of those parameters computed for all the samples are presented in Table 5.

**Figure 19:**
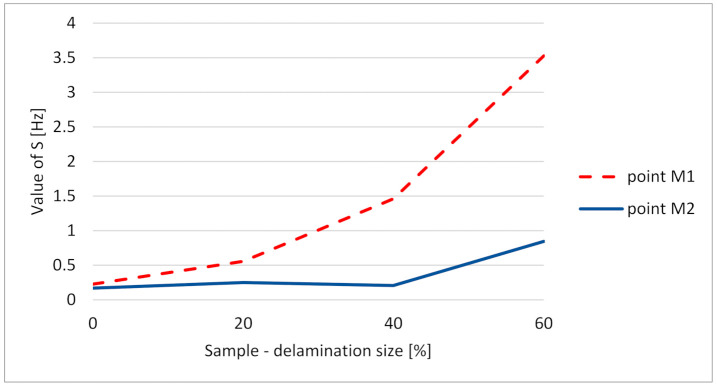
Standard deviation of vibration data for different delamination size.

For the performed measurements, the most informative parameters were standard deviation *S* and coefficient of variation *V*. Change of standard deviation between samples with increasing size of delamination is presented in Figure 19.

The experimental work confirmed periodic changes of weighted instantaneous frequency *f*_*w*_ caused by opening and closing of breathing delamination. Based on the measures like standard deviation, it is possible to build a failure indicator.

## 7. Conclusions

The paper discussed the application of the original Teager–Kaiser weighted instantaneous frequency $f_{w}$ dedicated for analysis of vibrational response of the beams with breathing delamination. In the framework of the presented research work:

- numerical models were created, reflecting breathing delamination phenomena in vibration signal;
- the conducted experimental tests confirmed half-period variation of instantaneous frequency resulting from breathing delamination;
- the weighted instantaneous frequency $f_{w}$, a new approach for estimation of instantaneous frequency, has been developed and its effectiveness was tested;
- a failure indicator and a diagnostic method were proposed for detection of breathing delamination type failure.

The main findings of the conducted research can be summarized as:

- the use of the Teager–Kaiser operator for the construction of the new weighted instantaneous frequency parameter gives much better prospects for delamination detection than in the case of use of instantaneous frequency obtained using demodulation with the Hilbert transform;
- standard deviation of $f_{w}$ can be a good indicator of delamination existence;
- the method is efficient for the analysis of the real-world vibrational signals, when proper signal processing is applied.

The performed research allowed to identify several limitations of the method, e.g., that the presence of noise in the signal causes that the calculation of $f_{w}$ requires additional signal processing steps such as averaging. Another drawback of $f_{w}$ is that it is based on the DESA-2 algorithm dedicated for demodulation of AM-FM signals. Despite the fact that the analyzed signal does not belong to AM-FM class, the method gives qualitative information on failure existence, although obtained results are overestimated. Another limitation of the potential SHM method, based on analysis of the $f_{w}$, is that excitation must allow delamination to open and close. In the considered case frequency, the first mode was used. It might be needed to refer to the FEM model of a structure to identify suitable excitation frequency.

The main benefit of $f_{w}$ is that, as long as the signal has high sampling frequency, short signals can be used for analysis because duration does not influence the result, as is in the case of integration-based methods. Based on the comparison of fluctuations of weighted instantaneous frequency for measurement points located in- and outside of the delamination area, one can deduce regarding delamination. Finally, it is important to stress that the main advantage of the proposed assisting damage detection approach is that it does not require analysis of the mode shapes of the structure, or any foreknowledge of the undamaged structure. This is for sure the key element that makes the method linked to the nature of the damage as such rather than to the structure.

The proposed approach can be useful for monitoring of simple beam-like structures e.g., composite bridge decks. However, the structure’s boundary conditions must allow for delamination breathing. This occurs for at least some of the boundary condition schemes, such as CF (clamped-free) or CC (clamped-clamped). As it was shown, in the case of a simple cantilever beam, the first mode was sufficient for delamination detection. In the case of more complicated structures, the proper excitation must be implemented to force breathing delamination phenomena to occur in all suspected components of the structure. In such a situation, multimodal testing can be necessary.

The experimental research presented in the paper was limited to the case of unidirectional arrangement of reinforcing layers in the specimens. In order to verify the method’s effectiveness for other stacking sequences, future research work will cover testing of specimens with various orientation of layers and the ratio of the stiffness of the separated part (upper sublaminate in Figure 7b) to the stiffness of the undamaged specimen. The mutual stiffness ratio will affect the amplitude of fluctuations of $f_{w}$ identifying the method’s applicability range.

The definition of the applicability scope of the method to other types of failures and development of the algorithm, allowing for identification of size of delamination, is an open issue and will be the subject of future work.

## Figures and Tables

**Table 1:** Statistical parameters of instantaneous frequency of the decaying signal.

**Decaying** **Factor** $A_{d}$	**Signal** **Duration** **[s]**	**Mean Frequency** **[Hz]**	**Maximum Frequency** **[Hz]**	**Minimum Frequency** **[Hz]**	**Standard Deviation of** **Frequency**
1	10	10.0162	11.3782	8.2789	0.9693
0.9	10	10.0155	11.6189	7.6486	0.9706
0.8	4.8994	10.0141	11.4111	7.2562	0.9704
0.7	2.3489	10.0032	11.4144	7.2461	0.9753
0.6	1.3455	9.9901	11.4354	7.2396	0.9805
0.5	0.8517	9.9698	11.3830	7.2315	0.9833
0.4	0.5732	9.9718	11.3523	7.2258	0.9824
0.3	0.4007	10.0263	11.3871	7.2232	0.9978
0.2	0.2855	10.0047	11.5053	7.2376	1.0157
0.1	0.2013	10.0841	11.7125	7.2946	1.0396

**Table 2:** Mechanical properties of the beam.

E_11_ [GPa]	E_22_ [GPa]	G_12_ [GPa]	ν_12_	ρ [kg/m ^3^]
17.4	17.4	1.45	0.3	2075

**Table 3:** Influence of the delamination size on weighted instantaneous frequency of the signals from delamination area (point “delam”) and from the free end (point “tip”).

$x_{a}/L$	Point	$\mathrm{Teager}–\mathrm{Kaiser}\;\mathrm{Weighted}\;\mathrm{Instantaneous}\;\mathrm{Frequency}\;f_{w}\;[\mathrm{Hz}]$			
		Mean	Maximum	Minimum	Standard Deviation
0.2	delam	25.915	26.328	25.469	0.204
	tip	25.993	26.293	25.743	0.153
0.3	delam	25.624	26.304	24.968	0.310
	tip	25.993	26.293	25.743	0.153
0.4	delam	25.147	26.691	23.525	1.065
	tip	24.995	25.451	24.663	0.192
0.5	delam	24.739	27.641	21.970	1.563
	tip	24.313	25.158	23.817	0.326
0.6	delam	20.658	24.784	16.526	2.651
	tip	22.949	24.041	22.258	0.401

**Table 4:** First modal frequencies for analyzed specimens.

**Specimen** **(** $x_{a}/L$ **Ratio)**	**Bending Modal Frequency [Hz]**					**Weight [g]**	**Average Width [m]**
	**1st**	**2nd**	**3rd**	**4th**	**5th**		
00—without delamination (0)	29.07	184.72	522.41	1014.36	1694.77	8.9	0.00143
20—with delamination (0.2)	30.07	184.73	499.66	1036.46	1534.63	8.8	0.00153
40—with delamination (0.4)	28.43	192.13	429.18	914.43	1451.81	9	0.00159
60—with delamination (0.6)	28.45	185.33	403.95	780.89	1320.15	9	0.00149

**Table 5:** Characteristic parameters of waveforms of instantaneous frequency for measurement points.

**Sample**	**Meas.** **Point**	$\bar{x}$ **[Hz]**	S **[Hz]**	V	D	$\frac{S_{M1}}{S_{M2}}$	$\frac{V_{M1}}{V_{M2}}$
00	M1	29.0347	0.2268	0.0078	0.824	1.3436	0.1345
	M2	28.9857	0.1688	0.058	0.707		
20	M1	29.9945	0.5557	0.0185	3.508	2.2192	2.2024
	M2	29.9481	0.2504	0.0084	1.169		
40	M1	28.4434	1.4609	0.0514	4.366	7.0780	7.0411
	M2	28.4013	0.2064	0.0073	0.704		
60	M1	27.2422	3.5267	0.1295	10.532	4.1781	4.3311
	M2	28.1925	0.8441	0.0299	2.886		

## Data Availability

The data presented in this study are available on request from the corresponding author.
